# Abstract, emotional and concrete concepts and the activation of mouth-hand effectors

**DOI:** 10.7717/peerj.5987

**Published:** 2018-12-07

**Authors:** Claudia Mazzuca, Luisa Lugli, Mariagrazia Benassi, Roberto Nicoletti, Anna M. Borghi

**Affiliations:** 1 Department of Philosophy and Communication, University of Bologna, Bologna, Italy; 2 Department of Psychology, University of Bologna, Bologna, Italy; 3 Department of Dynamic and Clinical Psychology, University of Roma “La Sapienza”, Rome, Italy; 4 Institute of Cognitive Sciences and Technologies, Italian National Research Council, Rome, Italy

**Keywords:** Type of concepts, Embodied and grounded cognition, Language processing, Abstract concepts

## Abstract

According to embodied and grounded theories, concepts are grounded in sensorimotor systems. The majority of evidence supporting these views concerns concepts referring to objects or actions, while evidence on abstract concepts is more scarce. Explaining how abstract concepts such as “freedom” are represented would thus be pivotal for grounded theories. According to some recent proposals, abstract concepts are grounded in both sensorimotor and linguistic experience, thus they activate the mouth motor system more than concrete concepts. Two experiments are reported, aimed at verifying whether abstract, concrete and emotional words activate the mouth and the hand effectors. In both experiments participants performed first a lexical decision, then a recognition task. In Experiment 1 participants responded by pressing a button either with the mouth or with the hand, in Experiment 2 responses were given with the foot, while a button held either in the mouth or in the hand was used to respond to catch-trials. Abstract words were slower to process in both tasks (concreteness effect). Across the tasks and experiments, emotional concepts had instead a fluctuating pattern, different from those of both concrete and abstract concepts, suggesting that they cannot be considered as a subset of abstract concepts. The interaction between type of concept (abstract, concrete and emotional) and effector (mouth, hand) was not significant in the lexical decision task, likely because it emerged only with tasks implying a deeper processing level. It reached significance, instead, in the recognition tasks. In both experiments abstract concepts were facilitated in the mouth condition compared to the hand condition, supporting our main prediction. Emotional concepts instead had a more variable pattern. Overall, our findings indicate that various kinds of concepts differently activate the mouth and hand effectors, but they also suggest that concepts activate effectors in a flexible and task-dependent way.

## Introduction

When we process and recognize words, do we activate the body? Do different kinds of words, as abstract, concrete and emotional words, activate different effectors, such as the mouth and the hand? Is this eventual activation modulated by the task?

The past years have seen the spread of embodied and grounded (from now on grounded) theories of cognition (Barsalou, 2008; Barsalou, 2010; Barsalou, 2016; Glenberg, 2015; Glenberg, Witt & Metcalfe, 2013; Borghi & Caruana, 2015), according to which concepts and words activate our bodily interactions with the world. Compelling evidence has demonstrated that when we hear words as for example ‘ball’ we re-enact previous interactions with the word referent, activating the sensorimotor system. This has been clearly demonstrated for words that refer to object or actions (Cappa & Pulvermüller, 2012; Glenberg & Gallese, 2012). While it is now established that concrete words are grounded in perception and action systems, the game is still open for abstract concepts and the words that express them, such as “fantasy” and “beauty”.

Explaining how not only concrete but also abstract concepts are grounded in the sensorimotor system represents a major challenge for embodied and grounded views, as recent debates testify (for recent reviews, see Borghi et al., 2017; Pecher, Boot & Van Dantzig, 2011; Wang et al., 2017; for special topics see Tomasino & Rumiati, 2013; Dove, 2015; Mahon & Hickok, 2016; Bolognesi & Steen, in press; Borghi et al., 2018a).

Compared to concrete words, abstract words typically lack a single object as referent, and refer to complex events and situations; furthermore, they are more detached from the five sensorial modalities, and are represented in a more variable way both across participants and within the same participant. Finally, according to recent proposals they are generally more grounded in internal states (interoception, metacognition, proprioception) Barsalou, 2003; Barsalou, Dutriaux & Scheepers, 2018; Borghi et al., 2018b; Borghi & Binkofski, 2014; Connell, Lynott & Banks, 2018; Kousta et al., 2011).

Two major novelties characterize recent literature on abstract concepts.

The first novelty is represented by the recognition that abstract concepts are not a monolithic whole, but that there might exist sub-kinds of abstract concepts that are differently represented. Recent studies have started to explore the differences between abstract concepts such as mathematic ones, emotional ones, mental states ones, social concepts, temporal concepts (e.g.,  Setti & Caramelli, 2005; Ghio, Vaghi & Tettamanti, 2013; Roversi, Borghi & Tummolini, 2013; Crutch et al., 2013; Mellem et al., 2016; C Villani, L Lugli, MT Liuzza, AM Borghi, 2018, unpublished data; Borghi et al., 2018b; Desai, Reilly & Van Dam, 2018). In this framework, it is debated whether emotions are to be considered as a sub-kind of abstract concepts or whether they differ from both concrete and abstract ones (for discussion see Mazzuca, Barca & Borghi, 2017; Barca, Mazzuca & Borghi, 2017). Altarriba, Bauer & Benvenuto (1999) and Altarriba & Bauer (2004) demonstrated that emotional concepts differ from concrete and abstract ones in ratings on a variety of psycholinguistics criteria, such as concreteness, imageability, and contextual availability, that they elicit different word associations, and that in free recall they are recalled better than concrete and abstract concepts. These authors have even argued that including emotional concepts among abstract concepts can lead to biased results (Altarriba, Bauer & Benvenuto, 1999).

For this reason, in the present work we did not include emotional concepts within abstract ones. Rather, we considered abstract, concrete and emotional concepts separately, in order to verify whether emotional concepts were responded to more similarly to concrete or to abstract concepts. We compared the processing of these three kinds of concepts in a lexical decision and in a subsequent word recognition task, measuring response times and accuracy. To investigate whether these concepts differently activate the hand/mouth effectors, in Experiment 1 participants were required to produce a response pressing a key either with the hand or with the mouth. In Experiment 2 participants were instead requested to respond using a pedal but had to keep a device either in their hand or mouth to respond to catch-trials. The first aim of the present study thus consists in comparing the processing of abstract, concrete and emotional concepts using response modalities that involve different effectors, i.e., the hand or the mouth, and two different tasks, a lexical decision task and a subsequent recognition task. Our intent is to verify whether abstract, concrete and emotional concepts are differently grounded in the sensorimotor system, and whether they differently activate the hand and mouth effectors.

The second novelty in current literature on abstract concepts is the emergence of multiple representation views (Borghi & Binkofski, 2014; Cuccio & Gallese, 2018; Dove, 2009; Dove, 2011; Dreyer & Pulvermüller, 2018; Kousta et al., 2011; Newcombe et al., 2012; Prinz, 2012; Recchia & Jones, 2012). These views represent an extension of grounded ones. They contend that, in order to fully account for abstract concepts representation, likely the linguistic, social, and emotional systems are activated beyond the perception and action systems.

Within multiple representation views, some proposals highlight the importance of language for abstract concepts representation. In particular, Dove (2014) has proposed that language is useful to improve our thought and our problem solving abilities (see also Clark, 1998; Lupyan & Clark, 2015; Dove, 2018). More relevant to the present work, the WAT (Words As social Tools) view (Borghi et al., 2018b; Borghi & Binkofski, 2014; Borghi et al., 2011; Borghi & Cimatti, 2009; Borghi & Zarcone, 2016) proposes that words are tools useful to operate in the physical and social environment. Specifically, abstract words would evoke linguistic and social experience more than other words. Abstract concepts are composed by sparse and heterogeneous exemplars, and linguistic labels would facilitate us in keeping these different exemplars together in a single category. Consistently with this perspective, literature on Modality of Acquisition (MoA) (Wauters, 2003) has shown that words acquired through the linguistic modality are more abstract and acquired later than words acquired through the perceptual modality, i.e., pointing to their referent (see also Thill & Twomey, 2016).

Studies by Topolinski and collaborators (Topolinski & Strack, 2009; Topolinski et al., 2014) have shown that during word reading we activate a simulation of the phono-articulatory aspects of words. According to WAT the phenomenon of covertly pronouncing words during reading is more pronounced when processing abstract words than concrete words. Of particular relevance for our hypothesis, previous findings suggest that abstract words elicit strong activations of brain areas linked with phonological processes and verbal short-term memory (Binder et al., 2005), and also a specific involvement of face motor areas in the processing of abstract mental nouns was observed (Dreyer & Pulvermüller, 2018). Recent lesion data are challenging the idea that these activations are just an epiphenomenal byproduct of the act of reading, supporting the idea that the motor system plays a causal role in the process of comprehending some kind of abstract-emotional words (Dreyer et al., 2015).

WAT proposes that the mouth motor system activation during abstract words processing is due to different mechanisms (see for extensive discussion Borghi et al., 2018b): the re-enactment of the linguistic experience associated to abstract concepts acquisition, the use of inner talk to re-explain to ourselves the word meaning, or a mechanism of “social metacognition”, consisting in the inner awareness of the inadequacy of our concepts, and in the need to ask others to help us. The simulation of word meaning would therefore play a substantial role for comprehension and not be simply a byproduct of word reading.

In sum: in line with the WAT view we contend that linguistic experience is particularly relevant for abstract concepts, because owing to their complexity and to the heterogeneity of their members they are difficult to acquire without the contribution of others. Because of such relevance of the linguistic experience, we hypothesize that during processing of abstract words the mouth motor system is involved, and thus that oral simulations are particularly crucial in the case of abstract words.

The second and more important aim of our study is to test the hypothesis that processing different kinds of words differently involves the mouth and the hand effectors. More specifically, we predict that the mouth motor system is more engaged for abstract than for concrete words, due to the fact that abstract concepts activate more linguistic experience. Consistently, previous evidence has shown that abstract words are rated as involving the mouth more than concrete words (Granito, Scorolli & Borghi, 2015), and that abstract sentences referring to mental states and to emotions are rated as involving the mouth more than math-related abstract sentences (Ghio, Vaghi & Tettamanti, 2013). Furthermore, behavioral evidence with response times has demonstrated that responses with the mouth were facilitated with abstract compared to concrete concepts in a definition-word-matching task (Borghi & Zarcone, 2016), and recent fMRI evidence has shown that abstract mental concepts evoke the mouth motor system (Dreyer & Pulvermüller, 2018).

We intend here to investigate whether the facilitation of mouth responses with abstract concepts, previously found in semantic tasks (e.g.,  Borghi et al., 2011; Granito, Scorolli & Borghi, 2015; Borghi & Zarcone, 2016), is present also in lexical decision task, and to verify whether it affects recognition. It is well known that in many cases semantic access is guaranteed by lexical decision tasks, but the processing level of lexical decision is shallower than that of semantic tasks (e.g., Barsalou, 2008). Notably, in fact, previous evidence on activation of effectors with verbs was obtained not only with sentences or verbs in evaluation tasks (e.g.,  Buccino et al., 2005), but also with passive reading tasks (e.g.,  Hauk, Johnsrude & Pulvermüller, 2004; Dreyer & Pulvermüller, 2018).

To investigate the involvement of the mouth effector in the processing of abstract words compared to concrete and emotional ones, we performed two different experiments. In Experiment 1 participants responded by pressing a button with the hand or with the mouth, in Experiment 2 they kept a button in the hand or in the mouth to respond to catch-trials, but responses to critical trials were given by pressing a pedal with the foot.

## Experiment 1

Previous results in which participants were required to decide whether a definition matched with a target words revealed that the processing of abstract concepts was facilitated with mouth responses, while the processing of concrete ones with manual responses (Borghi & Zarcone, 2016). In Experiment 1 we used the same response modality and the same response devices adopted by Borghi and Zarcone. We intended to verify whether the facilitation of abstract over concrete concepts in responses with the mouth was also present in a task involving a more superficial processing level, i.e., a lexical decision task, and in a subsequent recognition task. As to emotional words, we were interested in investigating whether they were processed similarly to other abstract words or whether they differed from both concrete and abstract words.

### Method

#### Participants

Forty native Italian speakers in a range of age between 20–30 years (22 females and 18 males; mean age: 20.1; standard deviation of age: 2.12) participated voluntarily. Handedness was assessed using an abridged version of the Edinburgh Inventory (Oldfield, 1971). All participants were Italian native speakers, had normal or corrected-to-normal vision, and were naïve as to the purpose of the experiment. All participants gave written informed consent, and the experimental procedures were approved by the Ethic Committee of the Institute of Cognitive Sciences and Technologies, Italian National Research Council, Rome, Italy (Approval number: 0000441).

### Materials

We selected 90 Italian words from the Della Rosa et al. database (Della Rosa et al., 2010), composed by 30 concrete words, 30 abstract and 30 emotional words. The selected words were balanced in Familiarity (mean = 590; *SD* = 148.09). We considered as concrete words the words that scored high on Concreteness (max. 700, min. 596; mean of Concreteness = 660.42; mean of Abstractness = 138.87), and considered as abstract the words with high Abstractness scores (max. 635, min. 375; mean of Abstractness = 535.24; mean of Concreteness = 213.28). We considered as emotional words those words that had intermediate scores of Abstractness and Concreteness (mean of Concreteness = 400; mean of Abstractness = 372.74) and that according to the experimenters had high emotional valence.

Because the emotional value was not present as a parameter in Della Rosa et al. (2010), we performed an on-line pre-test in which 25 participants (13 females; mean of age = 30.6; *SD* = 14.5) judged the emotional value of each word on a 7-points Likert scale (1 was rated as non-emotional and 7 as completely emotional). Since in the literature it is debated whether emotional words can be considered as a subset of abstract concepts or represent a kind of concept different from both concrete and abstract concepts (Altarriba, Bauer & Benvenuto, 1999; Kousta et al., 2011), the pre-test also aimed at clearly distinguish abstract, concrete and emotional words, to avoid overlaps between abstract and emotional words.

Among the original 90 words we selected 48 words: 16 more concrete, 16 more abstract and 16 rated as more emotional. Characteristics of the three categories of selected words in terms of dimensions and psycholinguistic variables are shown in Table 1(A).

**Table 1:** Characteristics of the three categories of words. A. Characteristics of the three selected categories of words in terms of psycholinguistic dimensions. B. Comparisons between the three selected categories of words in terms of psycholinguistic dimensions.

**Category**		Concreteness	Imageability	Familiarity	Age of acquisition	Context availability	Abstractness	Modality of acquisition	Number of letters	Emotional value	Frequency
**(A)**											
Abstract	Mean	232.28	261.37	441.27	469.27	357.96	530.64	566.43	9.25	2.90	70.25
	*N*	16	16	16	16	16	16	16	16	16	16
	*SD*	35.59	70.18	57.45	65.23	59.05	60.75	62.60	2.79	.57	63.28
Concrete	Mean	660.08	653.70	438.80	276.74	600.97	132.47	289.23	6.25	1.63	20.56
	*N*	16	16	16	16	16	16	16	16	16	16
	*SD*	30.90	47.99	57.32	81.84	57.96	28.53	91.12	1.65	.18	15.40
Emotional	Mean	321.49	346.32	438.57	395.73	436.12	446.51	483.73	8.13	5.20	82.69
	*N*	16	16	16	16	16	16	16	16	16	16
	*SD*	31.54	50.12	75.30	66.83	59.57	34.14	74.25	2.19	.52	61.92
Total	Mean	404.62	420.47	439.55	380.58	465.02	369.87	446.46	7.88	3.24	57.83
	*N*	48	48	48	48	48	48	48	48	48	48
	*SD*	188.96	179.21	62.56	106.52	117.45	178.31	139.46	2.54	1.56	57.56

**CATEGORY**	Concreteness	Imageability	Familiarity	Age of acquisition	Context availability	Abstractness	Modality of acquisition	Number of letters	Emotional value	Frequency
**(B)**										
Abstract-Concrete	*p* < .001; *t*(30) = − 36.27; *SE* = 11.79; *d* = 12.82	*p* < .001; *t*(30) = − 18.4; *SE* = 21.26; *d* = 6.52	*p* = .9	*p* < .001; *t*(30) = 7.3, *SE* = 26.17; *d* = 2.60	*p* < .001; *t*(30) = − 11.7; *SE* = 20.69; *d* = 4.15	*p* < .001; *t*(30) = 23.7; *SE* = 16.76; *d* = 0.92	*p* < .001; *t*(30) = 10.03; *SE* = 27.64; *d* = 3.54	*p* = .04; *t*(30) = 3.60; *SE* = 2.93; *d* = 1.27	*p* < .001; *t*(30) = 8.507; *SE* = .149; *d* = 3.068	*p* < .001; *t*(30) = 3.05; *SE* = 16.28; *d* = 1.07
Abstract-Emotional	*p* < .001; *t*(30) = − 7.49; *SE* = 11.92; *d* = 2.65	*p* < .001; *t*(30) = − 3.9; *SE* = 21.54; *d* = 1.39	*p* = .9	*p* = .004; *t*(30) = 3.1; *SE* = 23.35; *d* = 1.11	*p* < .001; *t*(30) = − 3.7; *SE* = 20.98; *d* = 1.31	*p* < .001; *t*(30) = 4.8; *SE* = 17.40; *d* = 1.70	*p* = .002; *t*(30) = 3.4; *SE* = 24.27; *d* = 1.20	*p* = .2	*p* < .001; *t*(30) = − 11.933; *SE* = .192; *d* = 4.294	*p* = .4
Concrete-Emotional	*p* < .001; *t*(30) = 30.60; *SE* = 11.06; *d* = 10.82	*p* < .001; *t*(30) = 17.7; *SE* = 17.35; *d* = 6.26	*p* = .9	*p* < .001; *t*(30) = − 4.4; *SE* = 26.45; *d* = 1.59	*p* < .001; *t*(30) = 7.9; *SE* = 20.79; *d* = 2.80	*p* < .001; *t*(30) = − 28.2; *SE* = 11.13; *d* = 9.97	*p* < .001; *t*(30) = − 6.6; *SE* = 29.37; *d* = 2.34	***p* = .02**; *t*(30) = − 2.32; *SE* = 0.68; *d* = 0.81	*p* < .001; *t*(30) = − 25.980; *SE* = .137; *d* = 9.391	***p* = .005**; *t*(30) = − 3.89; *SE* = 15.95; *d* = 1.37

We performed a *T*-Student test for independent samples by items and we calculated the Effect size (Cohen’s *d*), in order to verify if concrete, abstract and emotional words we chose differed in Concreteness, Abstractness and Emotional value.

All the categories (abstract, concrete and emotional words) resulted to differ in Concreteness (*p*_*s*_ < .001), Abstractness (*p*_*s*_ < 001) and Emotional value (*p*_*s*_ < .001). Emotional words were rated as more emotional than both abstract and concrete words (*p*_*s*_ < .001); also abstract words were rated as more emotional than concrete words (*p* < .001).

We also verified whether abstract, concrete and emotional words differed along a series of dimensions that, according to different theories, are considered crucial to distinguish concrete and abstract words, i.e., Imageability (IMG; Paivio, 1986), Age of Acquisition (AoA; Gilhooly & Logie, 1980), Context Availability (CA; Schwanenflugel, Akin & Luh, 1992) and Modality of Acquisition (MoA; Wauters et al., 2003). We decided to avoid controlling for these variables while selecting abstract and concrete words, because this would result in using a very reduced number of abstract and concrete concepts. More crucially, it would lead us to use concepts that are weird and scarcely familiar. We instead decided to select “good” abstract and concrete words: when compared to concrete words, “good” abstract words are typically less imageable (IMG), they are acquired later (AoA) and mainly through language rather than through perceptual modalities (MoA), and they are less associated to contexts (CA) (see e.g., Borghi et al., 2018b; C Villani, L Lugli, MT Liuzza, AM Borghi, 2018, unpublished data).

The abstract, concrete and emotional words we selected thus differed not only in Concreteness and Abstractness but also in Imageability, Age of Acquisition, Context availability, and Modality of Acquisition.

As to psycholinguistic variables, we took care in balancing words for Familiarity, thus the three categories didn’t differ on this dimension (all *p*_s_ = .9).

The abstract and emotional words we selected were more frequent than concrete ones (*p*_*s*_ < .05). We chose highly frequent abstract words because we wanted to avoid stimuli that were unfamiliar for participants which could have lead to abstractness driven effects. Emotional and abstract words did not differ for frequency (*p* = .4).

Concrete words were also shorter than abstract and emotional words (*p*_s_ < 04), while emotional and abstract words did not differ (*p* = .2).

Results of the comparisons between the three categories of selected words in terms of psycholinguistic variables and of all the relevant dimensions are shown in Table 1(B).

The effect of these variables on the results was determined through Generalized Linear Mixed Models (GLMMs) performed on both experiments (1 and 2) and tasks (Lexical Decision and Recognition) with accuracy and RTs as dependent variables. Together with *Type of Concept* and *Effector* as main factors, we added *Imageability*, *Age of Acquisition*, *Contex Availability*, *Modality of Acquisition*, *Frequency* and *Number of Letters* as covariates (see ‘Results’ section).

To complete our stimuli we subsequently added 48 pseudowords, created by modifying one letter at the beginning, in the middle or at the end of concrete, abstract and emotional words in the same proportion as the critical words. Then we created 24 words to be used as catch-trials: they were Italian words with a bold letter, at the beginning, in the middle or at the end of the word. Finally, we selected 24 new words, maintaining the proportion between abstract, concrete and emotional words for the recognition task. Words that can directly activate hand or mouth (e.g., tools or food related words) were excluded from the list. Stimuli are shown in Table 2.

**Table 2:** Selected stimuli from Della Rosa et al. (2010) database.

Italian word	English word	Frequency value	Number of letters	Frequency mean	N. letters mean
Affermazione	Affirmation	59	12		
Analogia	Analogy	8	8		
Circostanza	Circumstance	70	11		
Concetto	Concept	118	8		
Fascino	Appeal	149	7		
Funzione	Function	185	8		
Indiscrezione	Indiscretion	16	13		
Inefficienza	Inefficiency	17	12		
Inesperienza	Inexperience	5	12		
Insufficienza	Insufficiency	15	13		
Logica	Logic	107	6		
Merito	Merit	141	6		
Ozio	Idleness	9	4		
Reputazione	Reputation	27	11		
Tendenza	Tendency	161	8		
Unanimità	Unanimity	37	8	*Abstract*= 70.3	9.25
Alghe	Seaweed	24	5		
Alveare	Beehive	11	7		
Canoa	Canoe	25	5		
Circo	Circus	43	5		
Cravatta	Tie	38	8		
Elefante	Elephant	36	8		
Falce	Sickle	5	5		
Flotta	Fleet	35	6		
Gallo	Cock	12	5		
Giraffa	Giraffe	1	7		
Minerale	Mineral	9	8		
Oca	Goose	29	3		
Palude	Swamp	12	6		
Telegrafo	Telegraph	3	9		
Torre	Tower	44	5		
Trattore	Tractor	2	8	*Concrete*= 20.6	6.25
Abbandono	Abandonment	91	9		
Agitazione	Agitation	25	10		
Agonia	Agony	29	6		
Conflitto	Conflict	140	9		
Disperazione	Desperation	101	12		
Emergenza	Emergency	161	9		
Fallimento	Failure	99	10		
Fremito	Trembling	11	7		
Giuramento	Vow	13	10		
Impulso	Impulse	50	7		
Ira	Anger	45	3		
Orrore	Horror	77	6		
Pericolo	Danger	250	8		
Stupore	Wonder	62	7		
Terrore	Terror	96	7		
Tradimento	Betrayal	73	10	*Emotional*= 82.7	8.13

The experiment consisted of two tasks, a lexical decision task and a recognition task, that were presented in sequence; the lexical decision task always preceded the recognition one. Two separate lists of words were created for the two tasks: for the lexical decision task 24 critical words (8 concrete, 8 abstract, 8 emotional), and 24 pseudo-words. For the recognition task list, 24 critical words (8 concrete, 8 abstract and 8 emotional) and 24 new words were used.

### Procedure

**Figure 1:**
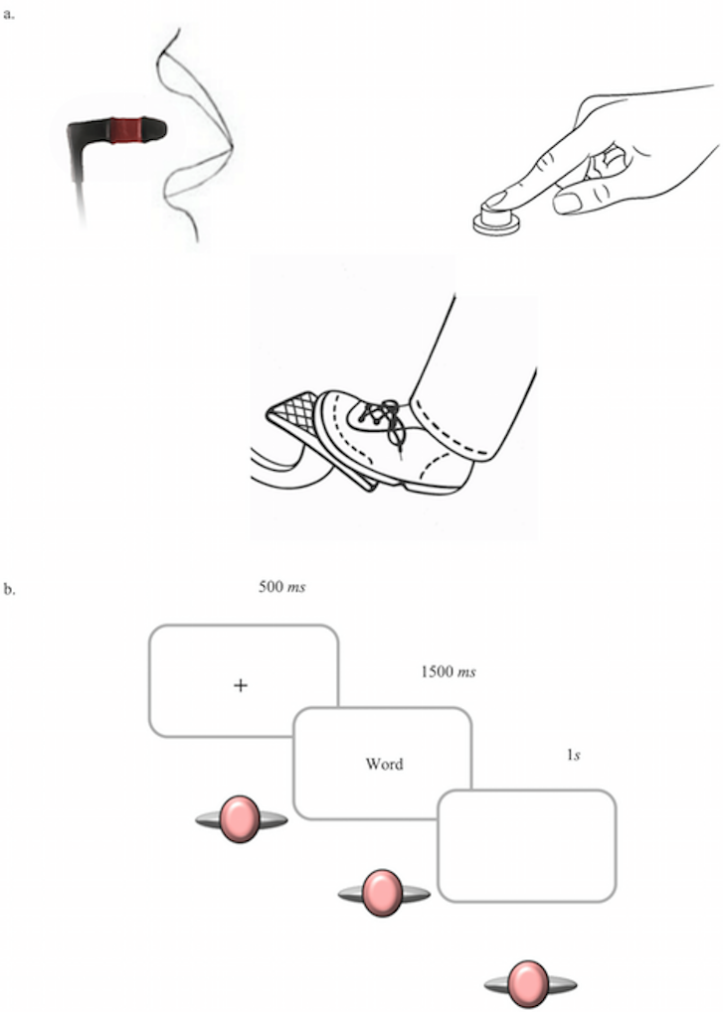
Procedure and devices used to respond to stimuli. (A) Mouth and hand buttons used to respond to stimuli in Experiment 1, and to catch-trial in Experiment 2; pedal used to respond to stimuli in Experiment 2. (B) Procedure: each trial began with a centred black fixation cross for 500 ms, followed by the presentation of the word. Words remained on the screen for a time of maximum 1.5 s. After 1 s the next trial started.

Participants were tested individually, and were instructed to respond as quickly and accurately as possible to each trial using a response box connected with a pedal and a button (see Fig. 1A). They were given the instructions on the computer screen and were trained at the beginning of every task. In no case further instruction from the experimenter was needed; she only needed to specify how to use the button for the mouth responses, and she made sure that participants used their dominant hand for hand’s responses. Participants were not aware of the subsequent recognition task; during the instructions they were just informed that they would complete an experiment composed by two phases. Only the participant and the experimenter were present in the room; after the training the experimenter sat outside the lab in order to avoid any kind of interference with the experiment. Testing took place on a Pc (resolution: 1,024 × 768 pixel) running EPrime2 Professional software. Participants sat on a comfortable chair in front of a computer screen, at a distance of about 60 cm.

Words appeared at the center of a computer screen at maximum 6.20° of visual angle. Each trial began with a centred black fixation cross for 500 ms, followed by the presentation of the word. Words remained on the screen for a time of maximum 1.5 s. After 1 s the next trial started (see Fig. 1B).

*Lexical decision task.* The task was divided into two experimental blocks, each preceded by a training block of 12 trials (6 words and 6 pseudo-words). Depending on the block, participants kept the button to be pressed with their dominant hand or with the mouth. The order of the blocks was counterbalanced across participants. A set of 48 words was presented on the computer screen (24 critical words, 24 pseudo-words). Participants were asked to press the button with the hand or with the mouth, depending on the instructions, if they read an Italian word, and to refrain from responding if the word they read was not an Italian one.

*Recognition task.* The task was divided into two experimental blocks, each preceded by a training block of 6 trials (3 words, 3 new words). Depending on the block, participants were required to keep the button in their dominant hand or in the mouth, between the teeth. A set of 48 words was presented in each block, composed by 24 critical words and 24 new words. The order of the blocks was counterbalanced across participants. Participants were asked to press the button with the hand or with the mouth, depending on the condition, in case they recognized the word on the screen as a word already presented in the previous task, or to refrain from responding if they read a new word.

### Results

The data were analyzed with Generalized linear mixed models (GLMM). Generalized linear mixed models (GLMM) incorporate random effects into the linear predictor of a generalized linear model (GLM). They include fixed linear predictor variables, within subject measures and random variances to account for cluster-related correlations in the data. GLMMs consider all the available data points, including those which occasionally failed on some trials. Furthermore, GLMMs can handle non-normal outcome distributions, which is important to account for accuracy, as it is likely to be highly skewed (Hedeker, 2005).

The effects of *Type of Concept* (abstract, concrete and emotional), of the *Effector* (mouth vs. hand) and of their interaction were analyzed on the response times and on the accuracy score (error) with two types of GLMM using *Frequency*, *Number of Letters*, *Imageability*, *Age of Acquisition*, *Context Availability* and *Modality of Acquisition* as covariates. We introduced these covariates to be sure that they did not affect our main results—for example, that the differences we found in processing the kinds of concepts were not due to a linguistic dimension or due to their different frequency.

In the first type of GLMM model, the response time was assumed to be normally distributed, therefore the Identity link function was used, whereas in the second type of model we adopted a logistic function because the accuracy was considered as having a binomial distribution (presence/absence of error). The two types of models were applied for the Lexical Decision and Recognition tasks of both Experiments 1 and 2.

Table 3 displays the GLMMs results in the different experiments showing the effect of *Type of Concept* (abstract, concrete and emotional) and the *Effector* (mouth vs hand) and their interaction as well as the covariates effect on the response time and accuracy.

Means of response times as a function of *Type of Concept* and *Effector* for both tasks (Lexical Decision and Recognition) and experiments (1 and 2) are displayed in Table 4.

*Lexical decision task.* All erroneous trials (2.96%) were removed before the analysis of RTs. In the lexical decision task, we found that the factor *Type of Concept* affected the response time [Wald (2) = 11.04; *p* = .004]. Abstract concepts (mean: 738.59 ms, *SE* = 10062.11) were responded to slower than emotional ones (mean: 697.27 ms, *SE* = 10058.79) and concrete ones (mean: 685.18 ms, *SE* = 10048.57). The analysis also showed an advantage for the hand (mean: 669.44 ms, *SE* = 10056.26) over the mouth effector (mean: 744.58 ms, *SE* = 10056.70) [Wald (1) = 77.43; *p* < .001].

**Table 3:** GLMMs results of Experiments 1 and 2 of both lexical decision and recognition tasks.

Experiment 1				
	Lexical Decision task		Recognition task	
Effects	RTs	Accuracy	RTs	Accuracy
Intercept	Wald(1) = 18.94; *p* < .001	Wald(1) = .35; *p* = .56	Wald(1) =23.8; *p* < .001	Wald(1) =15.1; *p* < .001
Type of Concept	**Wald(2) = 11.04; *p* = .004**	**Wald(2) =6.82; *p* = .03**	Wald(1) = .95; *p* = .62	Wald(1) = 3.73; *p* = .15
Effector	**Wald (1)= 77.43; *p* < .001**	Wald(1) = .12; *p* = .72	**Wald(1) = 22.68; *p* < .001**	Wald(1) = .54; *p* = .46
Type of Concept ×Effector	Wald(1) = .15; *p* = .92	Wald(1) = .38; *p* = .82	Wald(1) = 1.92; *p* = .38	**Wald(2) = 7.17; *p* = .03**
Number of Letters	**Wald(1) = 69.04; *p* < .001**	Wald(1) =3.64; *p* = .06	**Wald(1) = 25.17; *p* < .001**	**Wald(1) = 4.07; *p* = .04**
Frequency	**Wald(1) = 33.84; *p* < .001**	**Wald(1) = 11.07; *p* < .001**.	Wald(1) = 2.31; *p* = .13	**Wald(1) = 25.20; *p* < .001**
Age of Acquisition	**Wald(1) = 9.47; *p* = .02**	Wald(1) = 1.38; *p* = .24.	Wald(1) = 2.77; *p* = .96	Wald(1) = .69; *p* = .41
Imageability	Wald(1) = 1.98; *p* = .16	Wald(1) = .41; *p* = .52	Wald(1) = .65; *p* = .42	Wald(1) = 2.78; *p* = .10
Context Availability	Wald(1) = .60; *p* = .44	Wald(1) = .76; *p* = .38	Wald(1) = .92; *p* = .34	Wald(1) = .55; *p* = .46
Modality of Acquisition	Wald(1) = .23; *p* = .63	Wald(1) = 2.37; *p* = .12	Wald(1) = .102; *p* = .31	**Wald(1) = 6.18; *p* = .013**
**Experiment 2**				
Intercept	Wald(1) = 55.48; *p* < .001	Wald(1) = 6.04; *p* = .01	Wald(1) = 53.07; *p* < .001	Wald(1) = .74; *p* = .39
Type of Concept	**Wald(2) = 15.57; *p* < .001**	Wald(1) =2 .69; *p* = .29	**Wald(1) = 5.51; *p* = .06**	Wald(1) = 3.50; *p* = .17
Effector	Wald(1) = .69; *p* = .41	**Wald(1) = 4.88; *p* = .027**	Wald(1) = .64; *p* = .42	Wald(1) = 1.04; *p* = .31
Type of Concept ×Effector	**Wald(1) = 77.43; *p* = .08**	Wald(1) = 1.10; *p* = .57	**Wald(2) = 7.72; *p* = .02**	Wald(1) = 2.53; *p* = .28
Number of Letters	**Wald(1) = 33.68; *p* < .001**	Wald(1) = .04; *p* = .84	Wald(1) = .24; *p* = .62	Wald(1) =1 42; *p* = .23
Frequency	**Wald(1) = 10.83; *p* = .001**	**Wald(1) = 3.32; *p* = .068**	Wald(1) = .41; *p* = .52	Wald(1) = .88; *p* = .35
Age of Acquisition	**Wald(1) = 3.12; *p* = .078**	Wald(1) = .22; *p* = .63	Wald(1) = 2.12; *p* = .15	Wald(1) = .24; *p* = .72
Imageability	**Wald(1) = 7.38; *p* < .007**	**Wald(1) = 4.24; *p* < .039**	Wald(1) = 2.93; *p* = .09	Wald(1) = .78; *p* = .38
Context Availability	Wald(1) = 1.50; *p* = .22	Wald(1) = 1.61; *p* = .20	Wald(1) = .001; *p* = .97	Wald(1) = .56; *p* = .45
Modality of Acquisition	Wald(1) = 1.2569; *p* = .26	Wald(1) = .63; *p* = .43	Wald(1) = .009; *p* = .92	Wald(1) = .99; *p* = .32

**Table 4:** Means of response times as a function of Type of Concept and Effector for both tasks and experiments.

		RTs of Lexical Decision Task 1,2				RTs of Recognition Task 1,2			
		Experiment 1		Experiment 2		Experiment 1		Experiment 2	
Effector	Type of concepts	*M* (*RT*)	*SE*	*M* (*RT* )	*SE*	*M* (*RT* )	*SE*	*M* (*RT* )	*SE*
Mouth	Abstract	776.44 ms	10062.12	875.74 ms	10707.76	751.96 ms	14406.93	875.61 ms	11412.84
	Concrete	724.52 ms	10048.26	766.15 ms	10692.60	805.40 ms	14383.12	858.48 ms	11410.41
	Emotional	732.79 ms	10058.45	839.33 ms	10704.21	774.65 ms	14400.25	891.49 ms	11410.57
Hand	Abstract	700.74 ms	10062.10	876.55 ms	10707.97	721.65 ms	14404.91	901.59 ms	11415.72
	Concrete	645.83 ms	10048.90	777.19 ms	10692.41	750.50 ms	14384.32	852.80 ms	11413.15
	Emotional	661.76 ms	10059.14	804.56 ms	10703.91	705.84 ms	14398.77	844.32 ms	11412.07

All these effects are corrected for *Frequency*, *Number of Letters*, *Imageability*, *Age of Acquisition*, *Context Availability* and *Modality of Acquisition*, however only *Frequency* [Wald (1) = 33.84; *p* < .001; B = −.51], *Number of Letters* [Wald (1) = 69.04; *p* < .001; *B* = 18.36] and *Age of Acquisition* [Wald (1) = 9.46; *p* = .002; B = .31] affected the time of response in the lexical decision task. The predicted interaction between the two main factors did not reach the significance.

The factor *Type of Concept* also affected the accuracy of responses [Wald (2) = 6.82; *p* = .03]. The mean rates of errors corrected by the covariates were computed from the logistic model for each effect. Mean rates of errors are reported in terms of percentage of errors [abstract = 96%, *SE* = 13.15; concrete = 99%, *SE* = 4.87; emotional = 98%, *SE* = 5.38]. All the effects are corrected for the abovementioned covariates, however only *Frequency* [Wald (1) = 11.06; *p* = .001; B = .01] affected the accuracy of responses. No other main effect or interaction reached the significance.

*Recognition task.* All erroneous trials (28.30%) were removed before the analysis of RTs. In the analysis of response times, we found an advantage of the hand (mean: 726 ms, *SE* = 14405.92) over the mouth effector (mean: 777.34 ms, *SE* = 14396.75) [Wald (1) = 22.68; *p* < .001]. All the effects were corrected using *Frequency*, *Number of Letters*, *Imageability*, *Age of Acquisition*, *Context Availability* and *Modality of Acquisition* as covariates, however only *Number of Letters* [Wald (1) = 25.17; *p* < .001; *B* = 13.60] affected the response time in the recognition task. No other main effect or interaction reached the significance.

**Figure 2:**
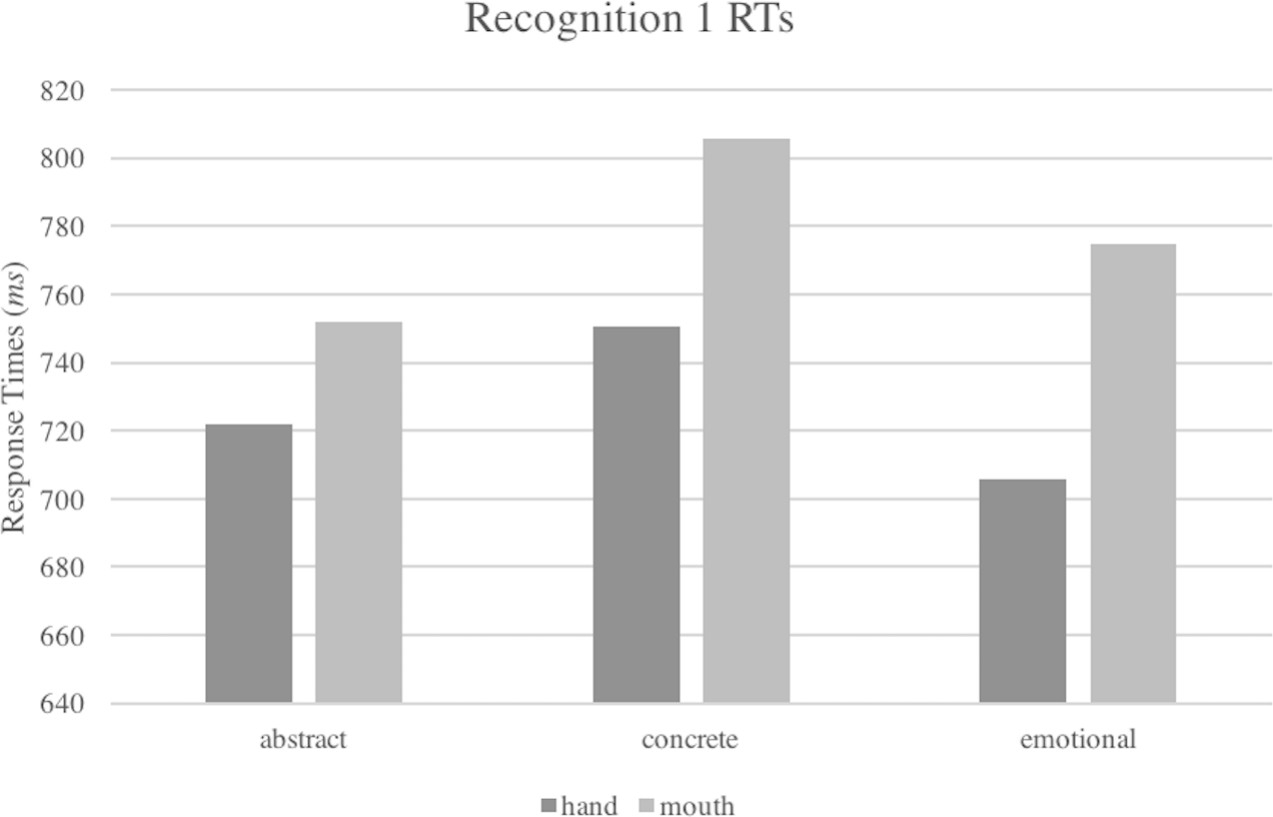
Interaction between *Type of Concept* and *Effector* factors in response times of Recognition, Experiment 1.

Nonetheless, a qualitative inspection of the interaction between the two main factors *Type of Concept* and *Effector* revealed that the pattern was in trend with our hypothesis: the advantage of the hand over the mouth responses was namely smallest with abstract words. Emotional words were the most affected by the effector, showing 70 ms of advantage of the hand over the mouth responses. They were followed by concrete words that showed a facilitation of 50 ms of the hand over the mouth responses, while abstract words showed the smallest advantage with the hand (30 ms of difference), as shown in Table 4 and Fig. 2.

**Figure 3:**
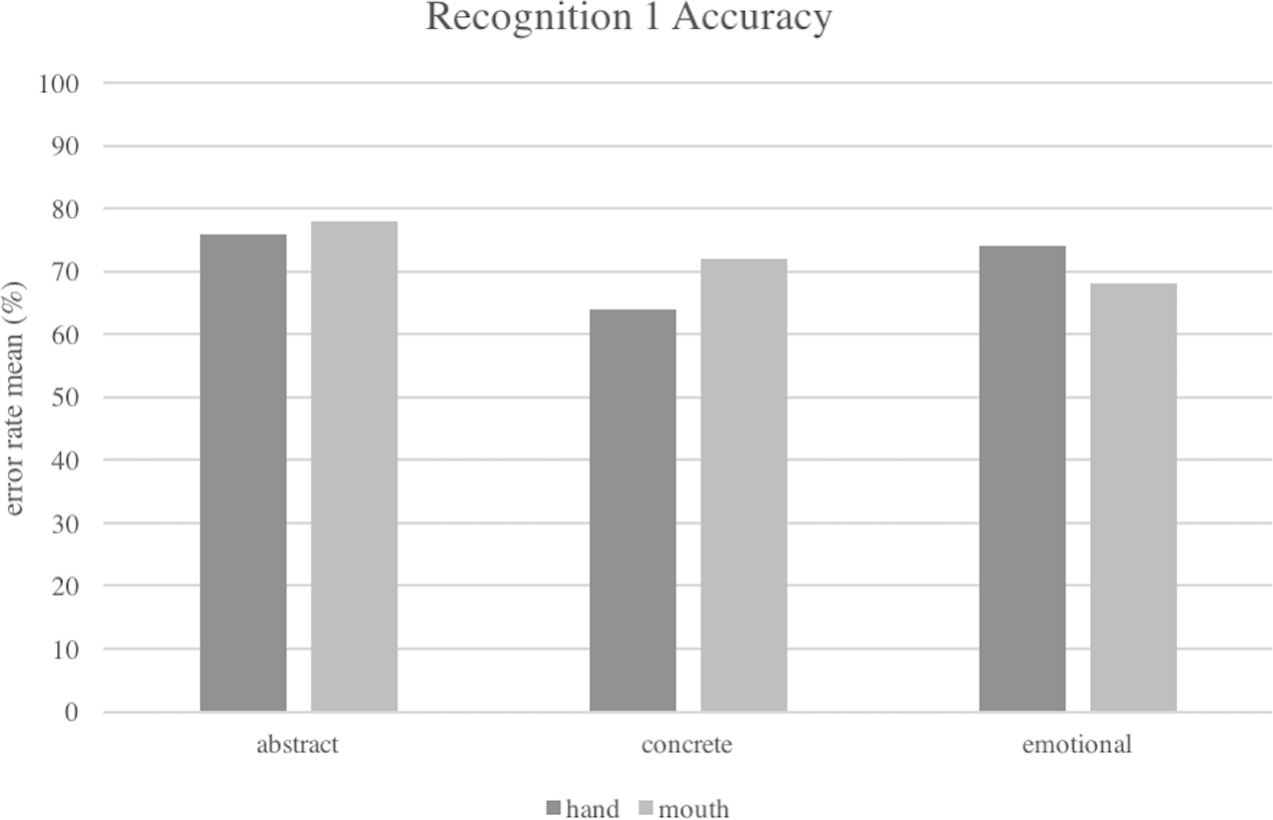
Interaction between *Type of Concept* and *Effector* factors in the accuracy of Recognition task, Experiment 1. The mean rate of errors were corrected by covariates included in the logistic model (*Frequency, Number of Letters, Imageability, Age of Acquisition, Context Availability and Modality of Acquisition*).

In the analysis on the accuracy, the interaction between the two main factors *Type of Concept* and *Effector* was significant [Wald (2) = 7.16; *p* = .02] (see Fig. 3). All the effects are corrected for the abovementioned covariates, however only *Number of Letters* [Wald (1) = 4.06; *p* = .04; B = −.05], *Frequency* [Wald (1) =25.20; *p* < .001; B = −.006] and *Modality of Acquisition* [Wald (1) = 6.17; *p* = .01; B = −.003] resulted to affect the accuracy. No other main effect reached the significance.

### Discussion

The results show that in the lexical decision task abstract words were processed slower than emotional words and in trend slower than concrete ones, confirming the well-established concreteness effect (Paivio, 1986). In the lexical decision task, *Frequency* and *Number of Letters,* together with *Age of Acquisition* appeared to be important variables, that may have affected the results since in general the selected abstract words were longer and acquired later. As to emotional words, their pattern seems to be similar to the pattern of concrete words: in general, they yielded shorter response times than abstract words, and they seem to be slightly advantaged in the hand condition.

Across the two tasks, responses with the mouth were slower than those with the hand, independently from the concept kind; this effect is not worth discussing since it was likely due to the fact that the button to hold among the teeth was harder to press than the key to press with the hand (see also Borghi & Zarcone, 2016).

As to the activation of the hand and mouth effectors in relation to the different type of concepts, in the lexical decision task we found no significant interaction. A possible explanation is that effectors are differently activated only in tasks that require a deeper processing level. The interaction between *Type of Concept* and *Effector* was instead significant in the recognition task, in the analysis on accuracy, in which the general disadvantage with the mouth effector was less pronounced with abstract words with respect to concrete ones, while emotional words showed an advantage with the mouth effector. It is worth noting that, beside *Frequency* and *Number of Letters*, *Modality of Acquisition* impacted significantly the results on accuracy of the recognition task. This could suggest that, in a task requiring a deeper level of processing the way in which we acquire words is more relevant than other psycholinguistic variables.

## Experiment 2

A potential problem of Experiment 1 was that the device used to respond to critical trials differed in the mouth and the hand conditions; this could explain why RTs were slower with the mouth responses. Experiment 2 was designed to verify whether the findings of Experiment 1 could be replicated also in an experiment in which the mouth and the hand were not the direct response effectors, but were nonetheless occupied during the task. We therefore introduced catch-trials, to which participants had to respond pressing the button either with the hand or with the mouth. Participants were instead invited to respond to critical trials by pressing a pedal with the foot, in order to avoid any potential interference with the hand and mouth effectors. This change had the advantage to allow us to manipulate the effector (hand vs. mouth) and at the same time to collect response times and errors with the same device, i.e., the pedal.

In this experiment we intended to test whether abstract, concrete and emotional concepts were differently activated when the mouth and the hand effectors were occupied. We predicted a facilitation of the mouth responses with abstract concepts.

### Method

#### Participants

Forty native Italian speakers in a range of age between 20–30 years (22 females and 18 males; mean of age: 23.5; standard deviation of age: 2.12) participated voluntarily. Handedness was assessed using an abridged version of the Edinburgh Inventory (Oldfield, 1971). All participants were Italian native speakers, had normal or corrected-to-normal vision, and were naïve as to the purpose of the experiment. All participants gave written informed consent, and the experimental procedures were approved by the CNR- ISTC ethics committee.

### Materials

Materials were the same as Experiment 1, except for 16 catch-trials that were added. Catch-trials were Italian words with a bold letter.

As in Experiment 1, the experiment consisted of two tasks, a lexical decision task and a recognition task, that were presented in sequence; the lexical decision task always preceded the recognition one. Two separate lists of words were created for the two tasks: for the lexical decision task 24 critical words (8 concrete, 8 abstract, 8 emotional) and 24 pseudo-words were used. For the recognition task list, the stimuli consisted of 24 critical words (8 concrete, 8 abstract and 8 emotional) and of 24 new words.

### Procedure

The procedure was the same as that of Experiment 1.

*Lexical decision task.* The task was divided into two experimental blocks, each preceded by a training block of 16 trials (8 words and 8 catch-trials). A set of 64 words was presented on the computer screen (24 critical words, 24 pseudo- words and 16 catch-trials). The words were arranged in two different lists, one for each block. Depending on the block, participants were required to keep a button in their dominant hand or in the mouth, between the teeth, and to respond to catch-trials by pressing it; they were instead asked to press the pedal to respond to critical stimuli. The order of the blocks was counterbalanced across participants. Participants were asked to press the pedal if they read an Italian word, and to refrain from responding if the word they read was not an Italian one. They were also required to respond to catch-trials by pressing the button with the hand or mouth, depending on the condition. Hence the mouth and the hand were not the direct response effectors, but depending on the condition either the mouth or the hand were occupied during the execution of the task.

*Recognition task.* The task was divided into two experimental blocks, each preceded by a training block of 8 trials (3 words, 3 new words and 2 catch-trials). A set of 62 words was presented in each block, composed by 24 critical words, 24 new words and 12 catch-trials. The order of the blocks was counterbalanced across participants. Depending on the block, participants were required to keep the button in their dominant hand or in the mouth, between the teeth. As to the critical stimuli, participants were asked to press the pedal in case they recognized that the word on the screen had already been presented in the previous task, or to refrain from responding if the read a new word. When catch-trials were presented, they had to respond by pressing the button with the hand or mouth, depending on the block.

### Results

*Lexical decision.* All erroneous trials (4.58%) were removed before the analysis of RTs. In the lexical decision task, the factor *Type of Concept* affected the response time [Wald (2) = 15.56; *p* < .001], showing that abstract words (mean: 876.14 ms, SE = 10707.86) were processed slower than both concrete (mean: 771.67 ms, *SE* = 10692.50) and emotional words (mean: 821.95 ms, *SE* = 10704.06). All the effects are corrected using *Frequency*, *Number of Letters*, *Imageability*, *Age of Acquisition*, *Context Availability* and *Modality of Acquisition* as covariates, however only *Frequency* [Wald (1) = 10.82; *p* = .001; B = −.33], *Number of Letters* [Wald (1) = 33.68; *p* < .001; *B* = 13.75] and *Imageability* [Wald (1) = 7.37; *p* = .007; B = .31] affected the response time. No other main effect or interaction reached the significance.

In the analysis on accuracy, the hand condition elicited more errors than the mouth condition. The mean rates of errors corrected by the covariates were computed from the logistic model for each effect. Mean rates of errors are reported in terms of percentage of errors (mouth: 95%, *SE* = 11.28; hand: 97%, *SE* = 7.09); (*Effector* [Wald (1) = 4.88; *p* = .02]. All the effects are corrected for the linguistic dimension of the stimulus, however only *Imageability* [Wald (1) = 4.24; *p* = .03; B = −.005] had an impact on the accuracy. No other main effect or interaction reached the significance.

**Figure 4:**
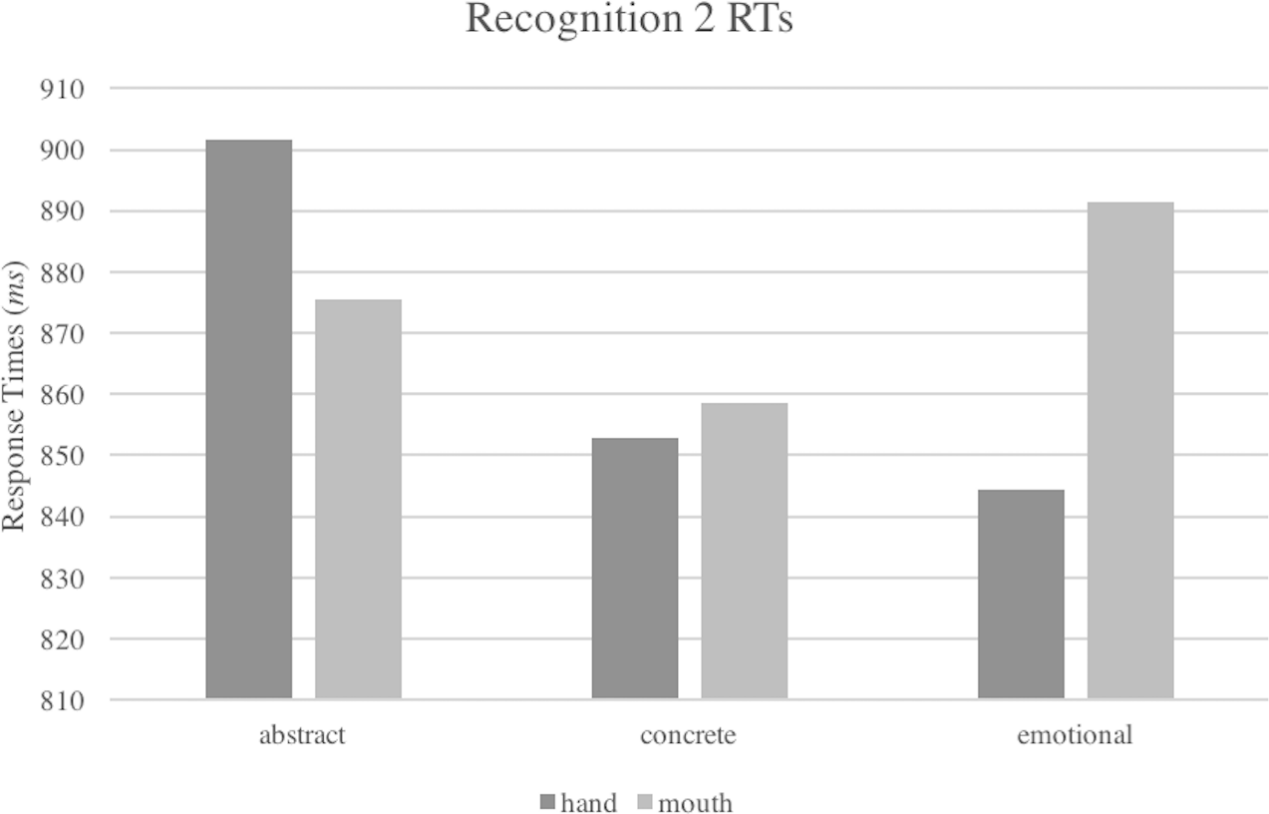
Interaction between *Type of Concept* and *Effector* factors in response times of Recognition task, Experiment 2.

*Recognition task.* All erroneous trials (26.1%) were removed before the analysis of RTs. In the analyses on response times, no main effect or effects of the covariates reached the significance. However, the interaction between the two main factors *Type of Concept* and *Effector* was significant [Wald (2) = 7.72; *p* = .02] showing the predicted interaction: abstract words were responded to faster with the mouth than with the hand, while concrete and emotional words were responded faster with the hand than with the mouth (see Table 4 and Fig. 4).

In the analysis on accuracy no main effect or interaction reached the significance, nor did the effect of the covariates.

### Discussion

Across the two tasks, abstract words were processed slower than both concrete and emotional words. As in Experiment 1, our findings confirm the concreteness effect and at the same time suggest that emotional words cannot be properly assimilated neither to concrete nor to abstract words. In line with the results of Experiment 1, we did not find a significant interaction between *Type of Concept* and *Effector* in the lexical decision task. The effect of the covariates *Frequency*, *Number of Letters* and *Imageability* in the lexical decision task, but not in the recognition task, suggests that these parameters could have affected more incisively a relatively shallow task, especially in the speed of processing. In the recognition task, instead, the interaction reached significance in the analysis of response times. Abstract words yielded faster RTs when processed in the mouth condition, while concrete and emotional words were responded to faster in the hand condition, as predicted by the WAT (Words As social Tools) view.

## General Discussion

According to some proposals, abstract concepts evoke more linguistic experience than concrete words (Borghi & Binkofski, 2014; Borghi et al., 2018b; Dove, 2011; Gleitman et al., 2005). If the involvement of language activates a motor simulation, this should result in a higher engagement of the mouth effector in the processing of abstract words, compared to that of concrete words. The main aim of this paper was to test the hypothesis that different kinds of concepts, i.e., concrete, abstract and emotional ones, differently engaged the mouth and hand effectors. In the two experiments we reported the mouth and the hand effectors were involved either directly, to provide a response (Experiment 1), or indirectly, keeping them occupied during the response (Experiment 2).

A further aim of this paper was related to the distinction between abstract, concrete and emotional words. Since many authors consider emotional concepts as a subset of abstract concepts while others tend to consider them as independent from both abstract and concrete ones, we intended to verify whether the performance with emotional words reflected that with abstract words or not.

Overall, our results confirmed the hypothesis that abstract words involve the activation of the mouth, but the effect was modulated by the task and differed depending on whether the response was provided directly using that effector or not. In the following we will point out the main results, discussing them in light of the advanced hypotheses. We will first illustrate results on the differences in processing the three concept kinds independently of the effector, then we will focus on differences between concepts kinds in relation to the activation of mouth and hand effectors.

### Overall processing differences between concept kinds

Overall, abstract concepts were processed generally slower than concrete ones in the lexical decision task (E1 and E2 lexical decision). Our results confirm the well-established concreteness effect (Paivio, 1986; but see exceptions to the effect when controlling stimuli for valence: Kousta et al., 2011; Barca, Burani & Arduino, 2002), that shows that abstract words are slower than concrete ones, and extended it showing that in the lexical decision task they are also slower than emotional words (see also Ponari, Norbury & Vigliocco, 2017). Interestingly, however, such effect reached significance only in the lexical decision task.

As to emotional words, our results cast doubts on the assimilation of emotional to abstract concepts: across experiments and tasks, the pattern of responses elicited by emotional words differed from that of abstract words and occasionally from that of concrete words too. In the lexical decision task of both experiments emotional words were processed faster than abstract words and did not significantly differ from concrete words. Our results are in line with those of a study by Siakaluk et al. (2016) showing that valenced words were processed faster than other words in lexical decision task. Overall, emotional words differed in processing from both concrete and abstract concepts, confirming the views according to which they represent a third kind of concept (Altarriba, Bauer & Benvenuto, 1999; Setti & Caramelli, 2005).

### Processing differences between concept kinds in relation to the effectors

The experiments we designed were driven from the hypothesis that abstract concepts would activate more the mouth motor system. Furthermore, we wanted to explore whether the two effectors, mouth and hand, would be differently activated with emotional words. We thus expected to find a *Type of Concept x Effector* interaction.

If we consider lexical decision, in neither experiment was the predicted *Type of Concept x Effector* interaction significant. Results thus seemed to suggest that the lexical decision task did not lead to a differential activation of the hand and mouth effectors depending on the concept kind, likely because of the superficial processing level it implied. This interpretation is supported by the fact that in the lexical decision task typical psycholinguistic variables such as *Frequency* and *Number of Letters* resulted to impact the speed of responses in both Experiments and also the accuracy in Experiment 1.

The results consistently differed if we consider the Recognition task. In Experiment 1 the interaction reached significance in accuracy and was present in trend in RTs, while in Experiment 2 the interaction was significant in RTs. In Experiment 1, the general disadvantage caused by the mouth device was smaller for abstract words with respect to concrete words in both the accuracy and the response times. In Experiment 2, in which the hand and mouth were occupied but responses were provided in the same manner, namely pressing a pedal with the foot, responses to abstract words were faster in the mouth than in the hand condition, while the opposite was true for both concrete and emotional words. These results clearly confirm our hypothesis, indicating that abstract words were facilitated when the mouth was activated, and extend previous results, showing that such a facilitation occurred not only when the mouth was the direct response effector but also when the mouth was occupied with a device.

This confirms the predictions of the WAT proposal, according to which abstract concepts re-enact linguistic and social experience more than concrete concepts, hence determining a higher activation of the mouth. Three possible mechanisms can underline this activation (see for further discussion Borghi & Zarcone, 2016; Borghi et al., 2018b): (a) the re-enactment of the acquisition experience, which is mainly linguistic and occurs in a social context; (b) the inner speech used to re-explain to us the meaning of abstract concepts; (c) the meta-cognitive awareness that our conceptual knowledge is inadequate followed by the motor preparation to ask to others information on words meaning (social-metacognition, Borghi et al., 2018b). The present study does not allow us to determine which of the three mechanisms is responsible of the effects; further research is needed in order to disentangle them.

The pattern of results of emotional concepts was also rather consistent across the two experiments, and clearly different from that of abstract concepts. In the recognition task of both Experiments 1 and 2, emotional words were processed slightly slower in the mouth than in the hand condition, differently from abstract words. Finally, the fact that abstract but not emotional words selectively engage only the mouth effector is in keeping with recent experimental results. Ratings results showed that emotional concepts activate both the mouth and the hand effectors, while mental states concepts activate more selectively the mouth (Ghio, Vaghi & Tettamanti, 2013). fMRI results clearly demonstrated that while the face/mouth motor system in the brain is more activated by “pure” abstract concepts as mental state concepts than by emotional ones, which activate hand and face motor cortex to similar degrees (Dreyer & Pulvermüller, 2018). Overall, this finding is in line with views according to which no strict dichotomy between abstract and concrete concepts exists. Our results rather suggest that emotional concepts cannot be assimilated neither to concrete nor to abstract concepts, (see Barca, Mazzuca & Borghi, 2017; Mazzuca, Barca & Borghi, 2017, for further discussion), and are in line with the proposal according to which emotional concepts, being more grounded than other abstract concepts, provide a bootstrapping mechanism to learn them (Ponari, Norbury & Vigliocco, 2017).

While we found that abstract concepts processing was facilitated with the mouth, the results are less marked than in a previous study (Borghi & Zarcone, 2016). We ascribe this difference to two factors: first, to the fact that in the previous study participants were provided with a context and not only with single words, and second, to the fact that the task was a deep processing one.

It can be objected that effectors effects have previously been found with lexical decision tasks. However, it is difficult to directly compare our results with those of previous studies with lexical decision, such as those by Pulvermüller (2005) and Neininger & Pulvermüller (2003), because these studies employed as stimuli action words directly related to the effectors (Pulvermüller, 2005) and strongly perceptual related words (Neininger & Pulvermüller, 2003).

Even if we did not find the predicted interaction with effectors, our findings are in line with those of previous lexical decision studies with concrete and abstract words. For example, Dreyer et al. (2015) comparing abstract emotional words with tool, food, animal words and effector related words, showed that in the control group abstract-emotional words yielded longer RTs than food and animal related words, while there was no difference in the accuracy. Moreover, in the same study (always in the control group), Dreyer et al. found that hand-related verbs were processed faster than verbs related to face and leg. Our results on lexical decision are in line with the evidence reported, showing a concreteness effect and a general advantage of responses given with the hand.

In sum, the present study adds important information to previous studies on concepts and effectors activation: it suggests that the mouth and hand effectors can be differently activated depending on the task and on the depth level it implies. The different effectors did not influence results in the lexical decision task, but they had an impact on a subsequent recognition task.

## Conclusion

Overall, our studies show that, in general, abstract words are more difficult to process than concrete ones, as revealed by the slower RTs, independently from the task. This confirms the concreteness effect, well-established in the literature. Furthermore, the pattern of results obtained with emotional words suggests that they are markedly different from both concrete and abstract concepts.

If we consider the relationship between concepts and effectors, we confirmed the hypothesis proposed by the WAT theory that abstract concepts had an advantage in the mouth condition. The result was however modulated by the task: the effectors did not have a different effect on concepts in a lexical decision task, but impacted a subsequent recognition task. Overall, our findings highlight that concepts are grounded and activate bodily experiences, but they also point out the exquisitely flexible character of our conceptual representation.

##  Supplemental Information

10.7717/peerj.5987/supp-1 Supplemental Information 1 Raw data of both tasks, Experiment 1 and 2, participants and materials

10.7717/peerj.5987/supp-2 Supplemental Information 2 Raw Data of Experiments 1 & 2 and SPSS syntax

## Additional Information and Declarations

### Data Availability

The following information was supplied regarding data availability: mazzuca, claudia; M. Borghi, Anna; Lugli, Luisa; Nicoletti, Roberto; benassi, mariagrazia (2018): Concept x Effector 1&2_R2. figshare. Fileset. https://doi.org/10.6084/m9.figshare.7146773.v1.
